# Gut viral metagenomics identifies viral signatures and their role in depression

**DOI:** 10.3389/fmicb.2025.1573851

**Published:** 2025-10-03

**Authors:** Xinyu Chen, Jun Wu, Dahua Fan, Peiwen Zhang, Yan Li, Yongkai Cao, Meiqun Cao

**Affiliations:** ^1^Graduate School of Guangxi University of Chinese Medicine, Nanning, China; ^2^Department of Neurology, Shenzhen Institute of Geriatrics, The First Affiliated Hospital of Shenzhen University, Shenzhen, China; ^3^Shandong College of Traditional Chinese Medicine, Shandong, China; ^4^Shunde Women and Children’s Hospital, Guangdong Medical University, Foshan, China; ^5^Daizhuang Hospital, Jining, China

**Keywords:** depression, gut virome, viral biomarkers, metagenomic sequencing, diagnostic signatures

## Abstract

**Background:**

The gut microbiome has been implicated in the onset and progression of depression. Yet, the role of the gut virome in depression remains unexplored, and a diagnostic model has not been satisfactorily constructed.

**Methods:**

Herein, we analysed the gut virome profiles of 29 patients with depression and 33 healthy controls using bulk metagenome sequencing.

**Results:**

A total of 45 differentially abundant viral taxa were identified, among which four, *s_Stenotrophomonas_virus_Pokken*, *g_Pokkenvirus*, *s_Dickeya_virus_AD1*, and *g_Alexandravirus*, demonstrated strong diagnostic potential (AUCs > 0.8). These four viruses also exhibited strong correlations, suggesting they may constitute a synergistic ecological cluster. Function annotation revealed seven metabolic pathways with significant differences, including alanine, aspartate, and glutamate metabolism, branched-chain amino acid (BCAA) biosynthesis, and energy metabolism in patients with depression.

**Conclusion:**

This study identified four distinct viral signatures for depression and proposes novel viral biomarkers for the diagnosis of depression, offering a robust diagnostic approach and new insights into the pathological mechanisms of depression.

## Introduction

Depression is a widespread and debilitating mental disorder, affecting approximately 280 million people worldwide (Vos et al., 2020). Its incidence significantly increased following the outbreak of COVID-19, underscoring the urgent need for more effective diagnostic strategies. In addition to its psychological impact, depression is also associated with an increased risk of developing cardiovascular diseases, Alzheimer’s disease, and cancer, contributing to increased mortality (Penninx et al., 2013). Despite its widespread prevalence and profound consequences, few robust diagnostic models and biomarkers have been identified for depression. Neuroimaging and genetic differences are often too subtle to distinguish patients with depression from healthy individuals.

Recent studies have highlighted the critical role of the gut microbiota in the onset and progression of depression (Schmidt et al., 2018). Viruses, as important components of the gut microbiota, may regulate depression through the gut-brain axis (Liang and Bushman, 2021; Cao et al., 2022). Therefore, the gut virome is highly important for understanding the pathogenesis of depression and identifying viral signatures for clinical diagnosis, yet it remains understudied. Yang et al. (2020) identified three differential bacteriophages: decreased *Clostridium_phage_phi8074-B1* and *Klebsiella_phage_vB_KpnP_SU552A* and increased *Escherichia_phage_ECBP5*. However, their diagnostic performance is limited, with an area under the curve (AUC) of 0.65 when two bacteriophages are combined. These findings underscore the need for further exploration of the virome’s role in depression pathogenesis.

In this study, we conducted a comprehensive analysis of the gut virome in a Chinese cohort comprising 29 depression patients and 33 healthy controls via bulk metagenome sequencing. A total of 45 differentially abundant viral taxa were identified through Wilcoxon and LEfSe analyses, from which four viral signatures were subsequently identified via logistic regression. Additionally, seven functional differences were found using STAMP and LEfSe analysis. Our findings provide new insight into the role of the gut virome in depression pathogenesis and offer promising biomarkers for the clinical prognosis of depression.

## Methods and materials

### General information

This study enrolled 29 patients with depression and 33 healthy controls recruited from Daizhuang Hospital, in Shandong Province, between January and December 2020.

### Inclusion and exclusion criteria

#### Diagnostic criteria

Depression was diagnosed using the Structured Clinical Interview for DSM-IV Axis I Disorders (SCID-I, Research Version). Depression severity was assessed using the 24-item Hamilton Depression Rating Scale (HAMD) and the Brief Psychiatric Rating Scale (BPRS).

#### Inclusion criteria

The participants in the depression group met the following conditions:

(1) Diagnosis of depressive disorder (first-episode or recurrent) based on DSM-IV criteria.
(2) HAMD score ≥20.
(3) Age between 15 and 60 years.
(4) No psychiatric medications (including antidepressants) or probiotics were used within the 12 weeks prior to enrollment.

The individuals in the healthy control group met the following conditions:

(1) No clinically evident symptoms of depression, with a HAMD score ≤7 or BPRS score ≤35.
(2) Age between 15 and 60 years.
(3) No psychiatric medications, including antidepressants, and no probiotic consumption within the 12 weeks before enrollment.

#### Exclusion criteria

The exclusion criteria were as follows:

(1) History of schizophrenia, substance abuse, or drug dependence.
(2) History of brain disease or endocrine disorders.
(3) Abnormal liver or kidney function.
(4) Pregnant or breastfeeding.
(5) History of manic or hypomanic episodes.
(6) Severe suicidal tendencies or a family history of psychiatric disorders.
(7) Recent inflammatory diseases or antibiotic use.

### Sample collection and processing

Fecal samples were collected from both healthy individuals and depressed patients. Total genomic DNA was extracted from the samples. The concentration, integrity, and purity of the genomic DNA were tested.

### Library construction and sequencing

Genomic DNA was fragmented, followed by end repair, adapter ligation, amplification, purification, and circularization to construct a single-stranded circular library. The constructed libraries were quantified using Qubit 2.0, and the fragment size was determined via the Agilent 2100 system. Accurate library concentrations (>2 nM) were verified via qPCR. The qualified library was pooled according to target data quantity and sequenced via the HiSeq platform (PE150) to generate raw reads in FASTQ format, which contained both sequence and quality information.

### Data processing and bioinformatics analysis

#### Data filtering and quality control

The raw sequencing data were filtered to remove reads containing more than 10% ambiguous bases (N bases), reads with adapter sequences (≥15 consecutive bases aligning to adapter sequences), or reads containing over 40% low-quality bases (*Q* < 20) via Trimmomatic (v0.38). The filtered data were further processed to remove host sequence (defined as reads with ≥90% similarity to the host genome) to minimize interference from host contamination in downstream analyses via the BMTagger.

#### Viral sequence assembly

After quality control and host sequence removal, MetaHIT was used to independently assemble the samples (k min: 21, k max: 149, k step: 10, m: 0.3). Contigs shorter than 200 bp were excluded to obtain valid assembled sequences.

### Statistical analysis

Statistical analyses were performed using R (v4.2.1) and STAMP software. Alpha and beta diversity analyses were performed via the vegan R package. Beta diversity was characterized using the Jaccard index and Bray–Curtis index. Demographic data, including sex and age, were analysed via the Wilcoxon rank-sum test. LEfSe analysis primarily involves three main steps: first, the non-parametric Kruskal–Wallis sum-rank test is employed to detect differences in taxa abundance across groups, thereby identifying taxa with significant differential abundance; second, the Wilcoxon rank-sum test is utilized to assess the consistency of these differences across subgroups within different groups; finally, linear discriminant analysis (LDA) is applied to estimate the magnitude of these differential abundant taxa, quantifying their contribution to group differentiation. Spearman correlation analysis was used to determine the correlation between differentially abundant viral taxa. Data visualization was performed via R (v4.2.1), STAMP, and GraphPad Prism (v9.3). Logistic regression analysis was conducted, followed by receiver operating characteristic (ROC) analysis to evaluate the diagnostic performance of the viral biomarkers. Logistic regression was performed using the glm() function in R with the default binomial family and no regularization. Variables were selected using stepwise backward elimination based on the Akaike information criterion (AIC).

## Result

### Demographic data

We conducted Wilcoxon rank-sum tests on the sex and age distributions between the healthy control (HC) group and the depression (DEP) group. The results revealed no significant differences in sex (*p* = 0.938) or age distribution (*p* = 0.544) between the two groups (Table 1).

**Table 1:** Clinical characteristics of the recruited subjects.

Group	Number of samples	Gender (female %)	Age	HAMD	BPRS
Healthy controls	33	66.7	35.67 ± 12.88	—	—
Patients with depression	29	65.5	34.27 ± 13.79	25.83 ± 6.59	36.52 ± 4.99
*p*-value	—	0.93	0.54	—	—

HAMD, Hamilton Depression Scale; BPRS, Brief Psychiatric Rating Scale.

### Viral community composition analysis

Through bulk metagenome sequencing, an average of 15.35 GB and 15.36 GB of raw data per sample were yielded for the HC and depression groups, respectively. After quality filtering and host sequence removal, the average number of reads per sample was 100,090,741 for the HC group and 98,946,115 for the depression group. Following viral sequence assembly, an average of 176,038 and 188,258 contigs per sample were generated for the HC and depression groups, respectively (see Table 2).

**Table 2:** Diagnostic potential of four viral signatures individually and in combination in training models.

Model No.	Viral signatures	AUC	Cutoff	Sensitivity	Specificity
1	s_Stenotrophomonas_virus_Pokken	0.832	0.65	0.889	0.667
2	g_Pokkenvirus	0.832	0.65	0.889	0.667
3	s_Dickeya_virus_AD1	0.921	0.498	0.852	0.958
4	g_Alexandravirus	0.897	0.609	0.852	0.917
5	s_Stenotrophomonas_virus_Pokken, g_Pokkenvirus	0.832	0.65	0.889	0.667
6	s_Stenotrophomonas_virus_Pokken, s_Dickeya_virus_AD1	0.916	0.484	0.852	0.958
7	s_Stenotrophomonas_virus_Pokken, g_Alexandravirus	0.904	0.621	0.852	0.917
8	g_Pokkenvirus, s_Dickeya_virus_AD1	0.916	0.484	0.852	0.958
9	g_Pokkenvirus, g_Alexandravirus	0.904	0.621	0.852	0.917
10	s_Dickeya_virus_AD1, g_Alexandravirus	0.924	0.492	0.852	0.958
11	s_Stenotrophomonas_virus_Pokken, g_Pokkenvirus, s_Dickeya_virus_AD1	0.916	0.484	0.852	0.958
12	s_Stenotrophomonas_virus_Pokken, g_Pokkenvirus, g_Alexandravirus	0.904	0.621	0.852	0.917
13	s_Stenotrophomonas_virus_Pokken, s_Dickeya_virus_AD1, g_Alexandravirus	0.917	0.481	0.852	0.958
14	g_Pokkenvirus, s_Dickeya_virus_AD1, g_Alexandravirus	0.917	0.481	0.852	0.958
15	s_Stenotrophomonas_virus_Pokken, g_Pokkenvirus, s_Dickeya_virus_AD1, g_Alexandravirus	0.917	0.481	0.852	0.958

In the analysis of the multiple-level viral composition, the following five most prevalent viral genera were detected: *Toutatisvirus*, *Taranisvirus*, *Mushuvirus*, *Lughvirus*, and *Punavirus*. These genera primarily include phages that target *Faecalibacterium* and other gut-associated bacteria. At the species level, the most dominant viral species included *Faecalibacterium virus Toutatis*, *Faecalibacterium virus Lugh*, *Faecalibacterium virus Oengus*, *Faecalibacterium virus Mushu*, and *Faecalibacterium virus Taranis*. Several members of the *crAssphage* group, such as *crAssphage cr4_1*, *crAssphage cr8_1*, and *crAssphage cr7_1*, were also consistently abundant across samples (Supplementary Figures 2, 3). These results suggest that *Faecalibacterium*-infecting phages and *crAssphage* viruses dominate the gut virome in both groups (Supplementary Figure 2).

**Figure 1:**
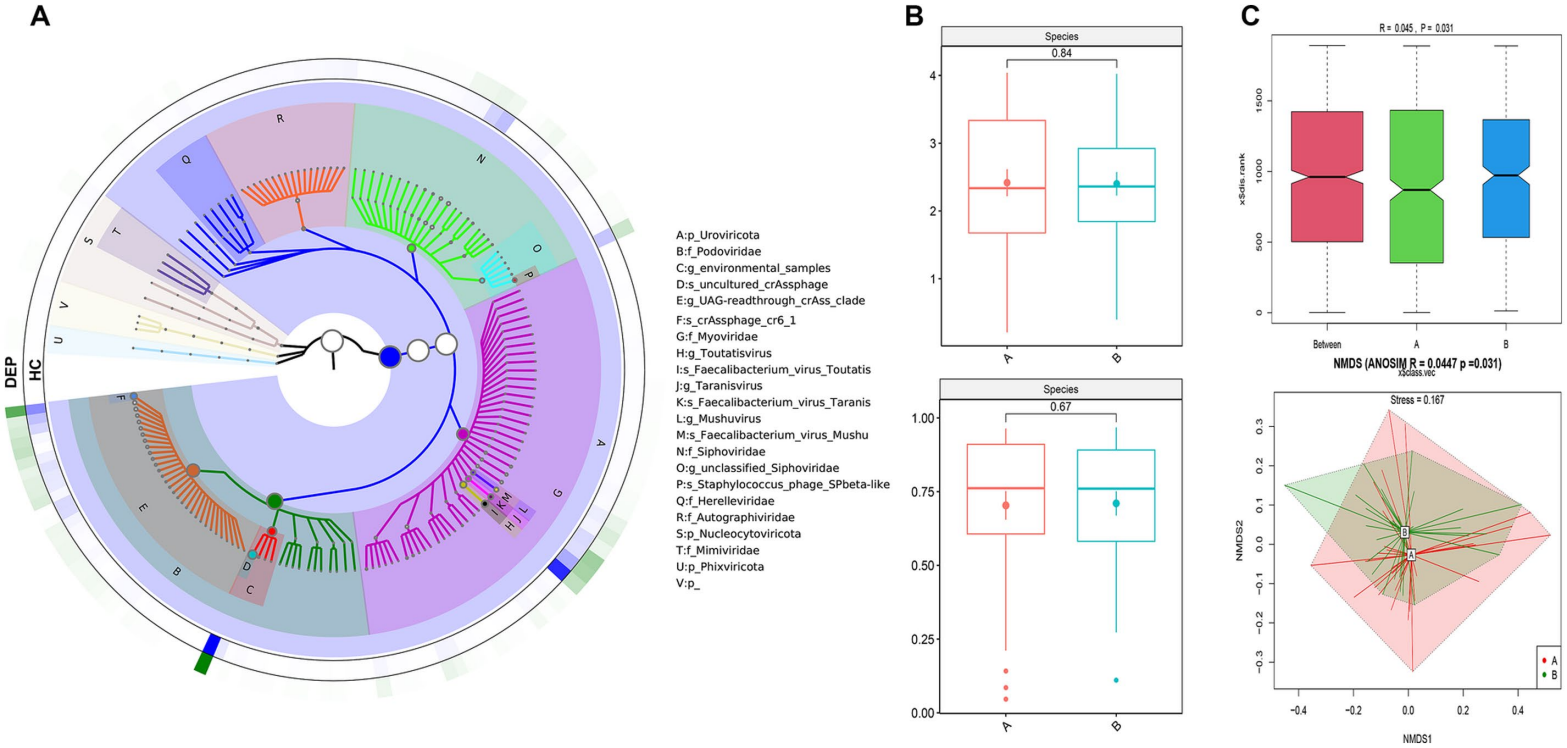
Viral composition and diversity of HC and DEP patients. **(A)** GraPhlan plot illustrating the hierarchical taxonomic composition of the gut virome in healthy control (HC) and depression (DEP) groups. The concentric circles represent different taxonomic levels (e.g., phylum, family, genus, species), with nodes representing specific viral taxa. Colors may differentiate between groups or highlight specific taxa. This plot visually depicts the relative abundances and structural differences of the virome between the two groups. **(B)** α-diversity analysis at species level: the upper panel displays Shannon analysis, and lower panel shows Simpson analysis. **(C)** β-diversity analysis at species level: the upper panel shows the ANOSIM (analysis of similarities) results, and the lower panel displays the NMDS (non-metric multidimensional scaling) ordination plot.

The GraPhlAn plot illustrates the hierarchical taxonomic composition of the gut virome in the normal control (HC) and depression (DEP) groups (Figure 1A). The results revealed differences in the distribution of viral abundances at different taxonomic levels between the two groups. Specifically, the depression group presented an increased relative abundance of *Uroviricota* at the Phylum level and *Myoviridae* at the family level, while the HC group presented increased abundances of *Phixviricota* at the phylum level and *Podoviridae* at the family level.

### Viral community diversity analysis

The rarefaction curves for observed species, genus, and family levels in the virome data indicated the sequencing depth achieved for each sample and the completeness of the diversity captured (Supplementary Figure 1).

#### Alpha diversity analysis

We performed α diversity analysis of the gut virome. As shown in Figure 1B, the Shannon (*p* = 0.84) and Simpson indices (*p* = 0.67) did not reveal significant differences between the HC and DEP groups at the species level. The α diversity of virome showed similar results at the genus level (Supplementary Figure 2). These findings suggested that the overall richness and diversity of the gut virome may be comparable between the samples.

#### Beta diversity analysis

Using evolutionary relationships and abundance information, we calculated the β diversity to evaluate differences in viral community structure between the HC and DEP groups. At the species level, the ANOSIM analysis yielded an *R* value of 0.045 and a *p*-value of 0.031, indicating a statistically significant difference in viral composition between the two groups. The NMDS analysis revealed partial overlap in species composition between the HC and DEP groups, suggesting a modest but significant divergence in gut virome structure (*p* = 0.031, stress = 0.167; Figure 1C). At the genus level, the ANOSIM analysis showed a higher *R* value of 0.168 with a *p*-value of 0.001, indicating a clear and significant separation in viral community structure between HC and DEP. Consistently, the NMDS plot demonstrated distinct clustering of the two groups, supporting a pronounced difference in gut virome at the genus level (*p* = 0.001, stress = 0.156; Supplementary Figure 1).

### Differential viral community analysis

#### Differential viral communities based on the Wilcoxon test

Based on the relative abundance of reads, we compared the compositions of the viral communities between the HC and DEP groups to identify differential virome features. Among the top 100 most abundant viral reads, 53.03% were enriched in the HC group. Notably, *f_Podoviridae* was the most abundant differential viral family in the DEP group, while *f_Rountreeviridae* and *f_Guelinviridae* were exclusively enriched in the HC group.

#### Differential viral analysis based on LEfSe

**Figure 2:**
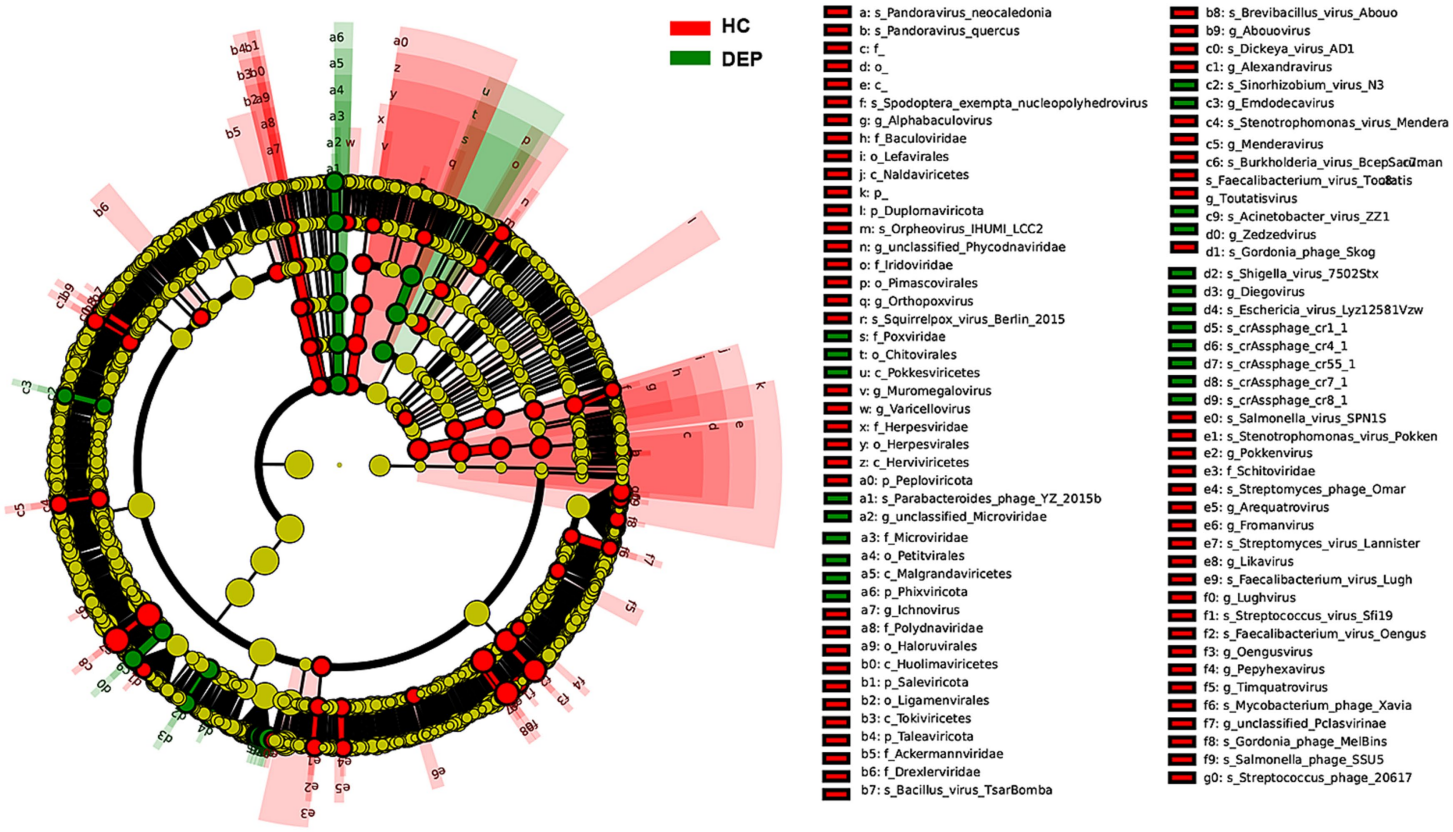
LEfSe analysis of differentially abundant viral species between the HC and DEP groups by cladogram. The inner circles represent higher taxonomic ranks (e.g., phylum, class), with branches extending to lower ranks (e.g., order, family, genus, species). Taxa enriched in the healthy control group are colored green, while those enriched in the depression group are colored red. Yellow nodes indicate taxa that are not significantly different between the two groups. The brightness of each node is proportional to its effect size (LDA score).

Using linear discriminant analysis effect size (LEfSe), we identified significantly different viral taxa between the HC and DEP groups. Cladograms and LDA score bar plots revealed that the HC group was significantly enriched in taxa such as *species of crAssphage_cr1_1*, *crAssphage_cr4_1*, *crAssphage_cr7_1*, and members of the family *Microviridae* (including *f_Microviridae* and *g_unclassified_Microviridae*), as well as the phylum *Phixviricota*. In contrast, the DEP group was enriched in taxa such as species *Pandoravirus_neocaledonia* and *Orpheovirus_IHUMI_LCC2*, family *Baculoviridae*, genus *Alphabaculovirus*, and higher taxonomic ranks including phylum *Duplornaviricota* and class *Naldaviricetes*. These findings suggest a link between depression and the expansion of specific viral taxa (see Figure 2).

LDA scores further indicated that although both the HC and DEP groups harbored distinct viral signatures, the DEP group exhibited a broader distribution of enriched species, particularly large DNA viruses from the Naldaviricetes and Baculoviridae lineages. This pattern suggests that depression may be associated with increased viral complexity or shifts toward DNA virus dominance in the gut virome. Notably, multiple *crAssphage* species were abundant in the HC group, while large nucleocytoplasmic DNA viruses were dominant in the DEP group.

### Biomarker analysis

**Figure 3:**
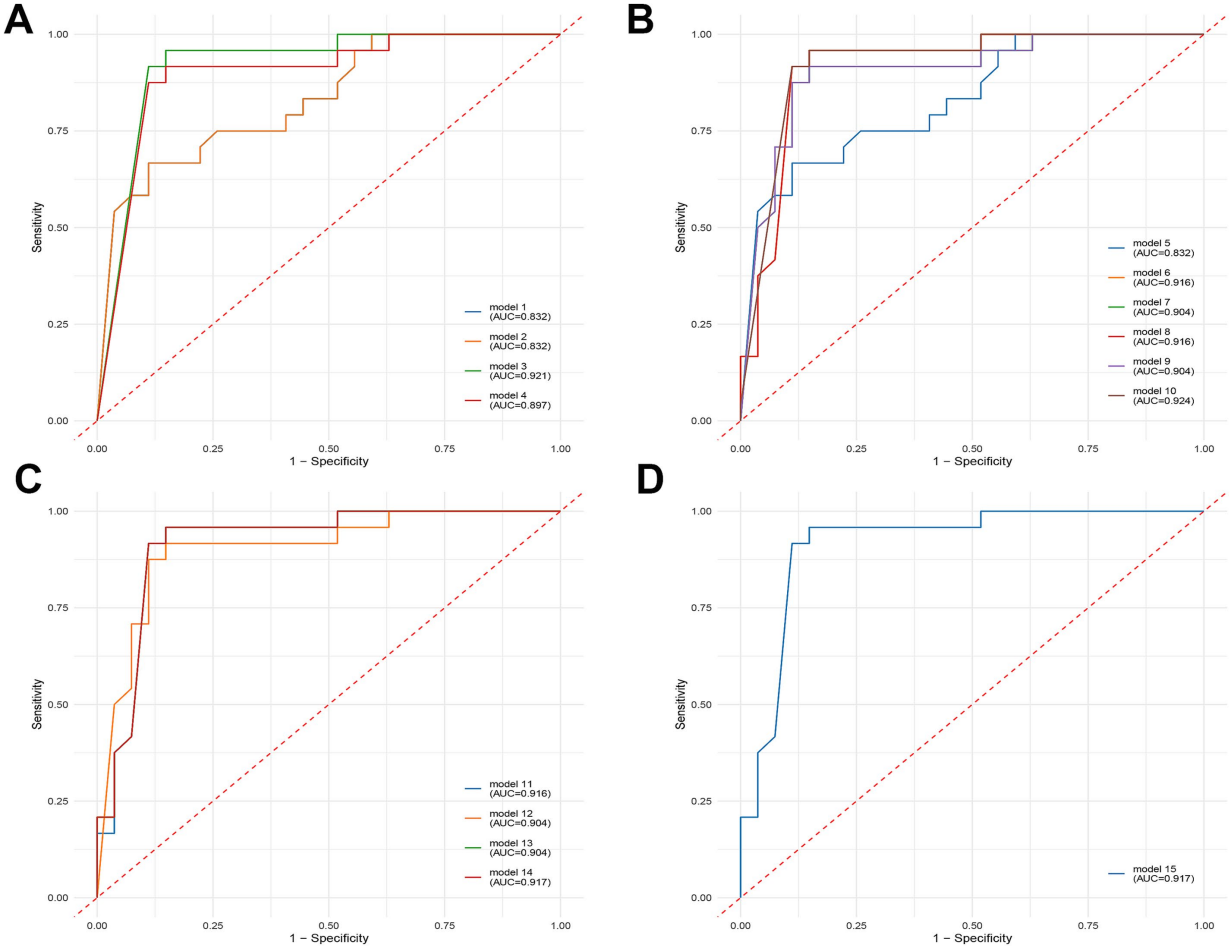
Diagnostic potential of selected viral signatures to differentiate between healthy controls (HC) and depression (DEP) patients, presented as receiver operating characteristic (ROC) curves with area under the curve (AUC) values. Each plot shows the true positive rate (sensitivity) against the false positive rate (1-specificity) for various models. Higher AUC values indicate better diagnostic accuracy. **(A)** Diagnostic performance of single-variable models. **(B)** Diagnostic performance of two-variable models, combining different pairs of viral signatures. **(C)** Diagnostic performance of three-variable models, combining different sets of three viral signatures. **(D)** Diagnostic performance of the four-variable model, combining all four selected viral signatures.

After Wilcoxon and LEfSe analyses, the viral taxa were further filtered by excluding those with zero values in more than 50% samples. This filtering yielded 45 candidate viruses. We then performed receiver operating characteristic (ROC) curve analysis using logistic regression to further screen the diagnostic signatures. Four individual viral features demonstrated strong classification performance, with AUC values exceeding 0.8 in the training set (Table 2). Specifically, species *Stenotrophomonas virus Pokken* and *Dickeya virus AD1* achieved AUCs of 0.832 and 0.921, respectively, while *genus Alexandravirus* achieved an AUC of 0.897 (Figure 3). Consistent performance was observed in the validation set.

To further enhance predictive accuracy, we constructed diagnostic panels by combining viral features in pairs, triplets, and as a full quartet. Using a five-fold cross-validation strategy with leave-one-out evaluation, we assessed each panel’s diagnostic performance in both the training and validation cohorts. Panels combining three or more viral features consistently exhibited robust performance, with some combinations achieving AUCs above 0.9 and maintaining balanced sensitivity and specificity. Notably, the panel composed of all four features (*s_Stenotrophomonas_virus_Pokken*, *g_Pokkenvirus*, *s_Dickeya_virus_AD1*, *g_Alexandravirus*) achieved an AUC of 0.917 in the training set and 0.867 in the validation set.

### Correlation analysis of differential viruses

**Figure 4:**
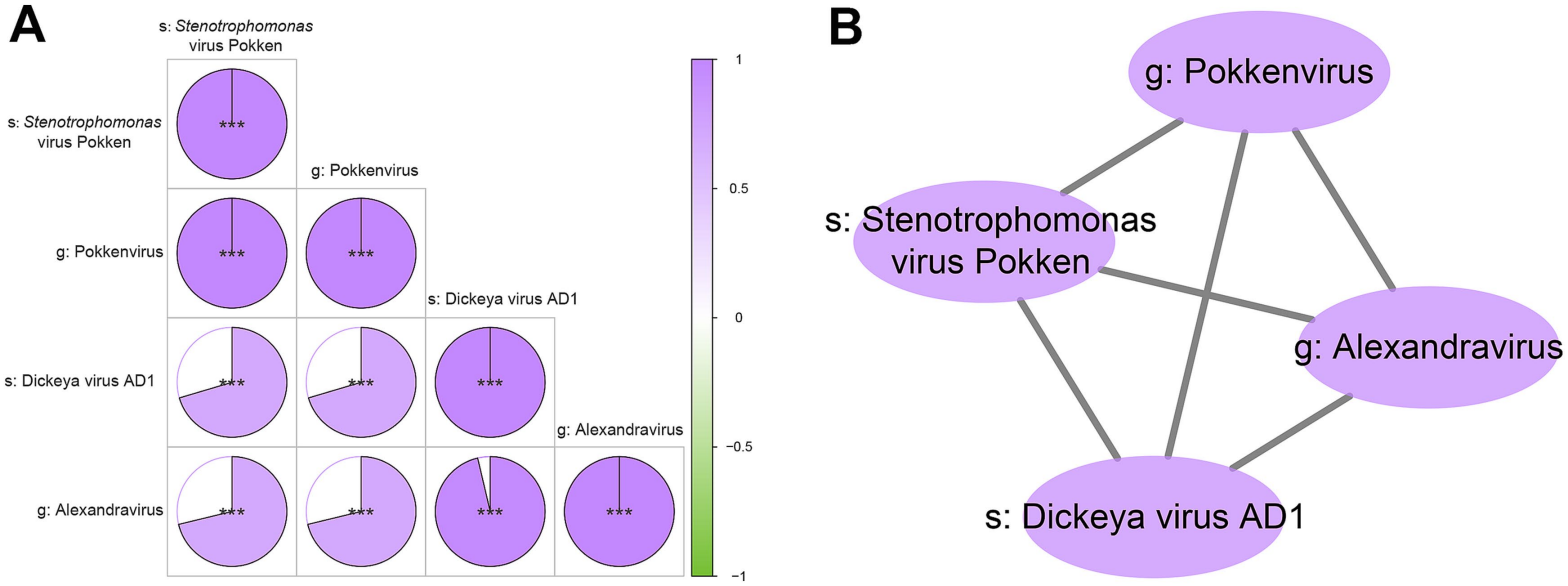
Intragroup correlation analysis and network visualization of the four key differential viral signatures. **(A)** Pie correlation chart of the four viral signatures. This plot displays the pairwise Spearman’s correlation coefficients between the relative abundances of the four viral signatures. Positive correlations are depicted by segments filling clockwise from 12 o’clock position, while negative correlations are indicated by segments filling counter-clockwise. The intensity of the fill color signifies the magnitude of the correlation coefficient, with deeper shades representing stronger correlation. Statistical significance of the correlation is denoted by asterisks: “***” indicates *p* < 0.001. **(B)** Network diagram illustrating significant correlations among the four viral signatures. In this network, each node represents one of the four key differential viral taxa. Edges connect nodes that exhibit a significant correlation in their relative abundances. The thickness of the edges is proportional to the strength of the correlation, with thicker lines indicating stronger relationships.

Based on the selected differential viral signatures, we further performed Spearman correlation analysis to investigate potential associations among these four viruses. The results revealed strong positive correlations among *s_Stenotrophomonas_virus_Pokken*, *g_Pokkenvirus*, *s_Dickeya_virus_AD1*, and *g_Alexandravirus* (correlation coefficient *r* > 0.95, *p* < 0.001; Figure 4). This indicated that these viruses may form a co-occurring ecological cluster with potential functional relevance in the context of depression. The consistent and significant correlations suggest that these viruses may not act independently, but rather engage in synergistic interactions, potentially reflecting shared ecological niches or coordinated roles in virome-host interactions relevant to the depressive phenotype.

### Viral functional analysis

We annotated the viral genes from both groups using the KEGG database after genome assembly. The annotation highlighted the pathways associated with transport, ABC transporters, and amino acid-related enzymes (Supplementary Figure 3).

**Figure 5:**
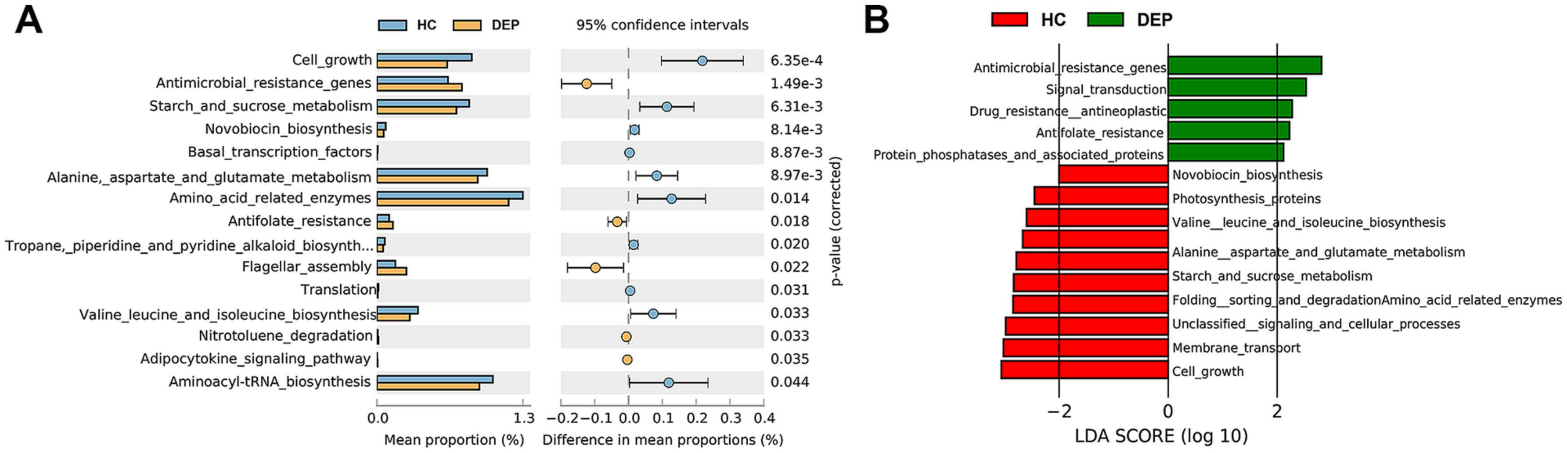
Analysis of differential functional pathways in the gut virome between HC and DEP groups. **(A)** STAMP analysis of differential functional pathways based on KEGG annotation. The x-axis represents the mean proportion of each functional pathway. The error bars indicate the 95% confidence intervals for these proportions. Pathways enriched in the HC group are shown in light blue, while those enriched in the DEP group are shown in light orange. The corrected *p*-values are also displayed, indicating the statistical significance of these differences. **(B)** Linear discriminant analysis (LDA) scores of differential functional pathways in either the HC or DEP group. Green bars extending to the right represent pathways enriched in the depression group, while red bars extending to the left represent pathways enriched in the healthy control group. The length of each bar is proportional to the LDA score, which quantifies the effect size of the differential abundance, highlighting the most influential functional pathways driving the distinctions between the two groups.

Furthermore, the differential functions of viruses were analysed via STAMP and LEfSe. Both analyses identified seven differential functional pathways between the HC and DEP groups. The DEP group presented elevated antimicrobial resistance genes and antifolate resistance pathways, suggesting increased microbial resistance in patients with depression. In contrast, pathways such as starch and sucrose metabolism, novobiocin biosynthesis, amino acid metabolism (e.g., alanine, aspartate, and glutamate), branched chain amino acid (BCAA) biosynthesis (e.g., valine, leucine, and isoleucine), and cell growth were significantly reduced in the DEP group. These findings indicate impaired amino acid biosynthesis and energy metabolism, and decreased metabolic activity in depressed patients (see Figure 5).

## Discussion

### Potential role of the gut virome in depression

In recent years, emerging evidence has indicated that disturbances in the gut virome may contribute to the development and progression of depression. Gut bacteriophages, key components of the gut virome, influence psychiatric disorders by releasing pathogen-associated molecular patterns (PAMPs), which activate inflammatory signaling pathways, affecting brain function (Columpsi et al., 2016). Additionally, gut viruses can contribute to the pathogenesis of depression and impact brain development and function by altering gut permeability, modulating host immune responses. This modulation can lead to the release of inflammatory mediators (cytokines) that can cross the blood–brain barrier, consequently disrupting the gut-brain axis (Li et al., 2021; Ritz et al., 2024). Specific viral signatures may therefore be associated with increased or decreased levels of pro-inflammatory cytokines, directly impacting neuroinflammation in depression (Duan et al., 2022).

Despite their important role in gut ecology, the association between the gut virome and depression remains underexplored. This study leverages the virome to elucidate key characteristics of the gut virome in patients with depression, providing novel insights into its involvement in depression.

Comparative analyses of the gut virome in patients with major depressive disorder (MDD) and healthy controls have identified significant differences in the abundance of certain bacteriophages (Yang et al., 2020), suggesting that gut virome dysbiosis may influence depression through microbiome disruption, neurotransmitter imbalance, or immune modulation. Similarly, animal models and viral transplantation further prove that restructuring the gut virome can reverse stress-induced behavioral and immune abnormalities (Ritz et al., 2024). A study integrating the virome and metabolomics revealed that chronic stress induces significant changes in the gut virome, particularly the enrichment of *f_Microviridae* viruses, which are closely associated with neurotransmitter metabolism. These differential viral taxa coincide with our identified viral family of *Microviridae* (Duan et al., 2022). These findings suggest that the gut virome may influence depression pathogenesis through the regulation of neurotransmitter metabolism and immune responses.

HC group was significantly enriched in taxa such as *species of crAssphage_cr1_1*, *crAssphage_cr4_1*, *crAssphage_cr7_1*, and members of the family *Microviridae* (including *f_Microviridae* and *g_unclassified_Microviridae*), as well as the phylum *Phixviricota*. In contrast, the DEP group was enriched in taxa such as species *Pandoravirus_neocaledonia* and *Orpheovirus_IHUMI_LCC2*, family *Baculoviridae*, genus *Alphabaculovirus*, and higher taxonomic ranks including phylum *Duplornaviricota* and class *Naldaviricetes*.

### Changes in virome diversity and composition

This study found increased β diversity of the gut virome in depression patients, while no significant differences in α diversity. This finding indicates that the overall community structure of groups rather than individuals is more relevant to depression (Shkoporov et al., 2019). Notably, four diagnostic viral signatures were identified: *s_Stenotrophomonas_virus_Pokken*, *g_Pokkenvirus*, *s_Dickeya_virus_AD1*, and *g_Alexandravirus*. Of these, *s_Stenotrophomonas_virus_Pokken* and *g_Pokkenvirus* showed elevated abundance in the HC group, while the enrichment patterns for *s_Dickeya_virus_AD1* and *g_Alexandravirus* require further investigation to determine their specific direction of association with depression. In contrast, the HC group generally showed higher abundances of genus *crAssphage* and family *Microviridae*. These viruses may participate in depression pathogenesis by stabilizing gut microbiome stability, modulating host immune responses, and regulating metabolite production.

### The gut virome as potential biomarkers and potential mechanisms of depression

To date, few clinical studies have explored the potential of virome markers for depression diagnosis. Yang et al. (2020) identified *Clostridium_phage_phi8074-B1* and *Escherichia_phage_ECBP5* as diagnostic markers for depression, but with limited diagnostic performance (AUC = 0.65). In contrast, our study identified *s_Stenotrophomonas_virus_Pokken*, *g_Pokkenvirus*, *s_Dickeya_virus_AD1*, and *g_Alexandravirus*, as well as any of their combinations, demonstrated high diagnostic accuracy, with all AUC values greater than 0.8. Interestingly, the highest AUC reached 0.924, significantly outperformed previously reported diagnostic performances.

The viral species *Stenotrophomonas virus Pokken* is a bacteriophage that infects *Stenotrophomonas maltophilia*, and it is classified under the subspecies *Pokkenvirus pokken* (Brooke, 2012). *S. maltophilia* is an emerging multidrug-resistant Gram-negative bacterium associated with nosocomial infections, posing a serious threat to immunocompromised individuals and patients with cystic fibrosis, potentially leading to severe pulmonary diseases. Reports on *S. virus Pokken* itself as a disease biomarker are scarce; however, volatile metabolic compounds released by *S. maltophilia* have been proposed as potential biomarkers to distinguish this pathogen from other microorganisms involved in pulmonary infections (Brooke, 2021). In this study, the enrichment of its corresponding phage, *s_Stenotrophomonas_virus_Pokken*, in the healthy control group may reflect host bacterial ecology or phage-host equilibrium dynamics.

The genus *Pokkenvirus* includes the *Stenotrophomonas* phage Pokken, which may partially explain the consistent diagnostic performance and ROC curve trends observed between *g_Pokkenvirus* and *s_Stenotrophomonas_virus_Pokken*. The elevated abundance of this virus in the healthy group could be influenced by environmental or dietary factors affecting its colonization. However, current evidence does not support a direct association with depression, and further investigation is needed to clarify its taxonomic status and host specificity.

The viral species *Dickeya virus AD1* is a bacteriophage that infects the phytopathogen *Dickeya solani*, which is associated with wilting and soft rot diseases in potatoes and various ornamental and crop plants (Day et al., 2018). While jumbo phages infecting *D. solani* have been reported, there is currently no literature specifically identifying *Dickeya virus AD1* as a disease biomarker. Its detection in the gut virome may result from dietary intake of plant-based foods carrying its host bacterium, but the ecological significance of its differential abundance remains unclear.

The genus *Alexandravirus* primarily comprises phages that infect soft rot bacteria, including species within *Pectobacterium* and *Dickeya*. Members of this genus exhibit broad host specificity and genomic conservation and are capable of infecting Gram-negative bacteria. Notably, *Alexandravirus* phages have been shown to infect *Dickeya solani*. These bacteria are significant plant pathogens responsible for a wide range of plant diseases (Kim et al., 2022). The presence of *Alexandravirus* in the gut may reflect its regulatory potential over multiple bacterial hosts, although its specific targets and mechanisms of action require further elucidation.

### Functional insights from KEGG analysis

KEGG functional analysis in this study identified several virome-related pathways associated with depression, particularly those involving amino acid biosynthesis and metabolism, energy metabolism, and cell growth.

Glutamate, the primary excitatory neurotransmitter in the central nervous system, plays a key role in depression pathophysiology by contributing to inflammation and synaptic dysfunction (Haroon et al., 2018). Dysregulated glutamate levels may disrupt neurotransmitter systems, impairing emotional regulation and cognitive functions. Acute stress can elevate glutamate levels, causing neurotoxicity, whereas chronic stress reduces glutamate cycling and metabolism, correlating with weakened functional connectivity in the prefrontal cortex and mood dysregulation (Murrough et al., 2017; Duman et al., 2019). Therefore, this study revealed reduced glutamate metabolic pathway activity in the depression group, suggesting that the gut virome-mediated regulation of glutamate metabolism may mediate neuroinflammation, synaptic function, and neurotransmitter balance.

Valine, leucine, and isoleucine biosynthesis belong to BCAAs biosynthesis, which is essential for protein synthesis, energy balance, and neurotransmitter regulation (Tian et al., 2022; Shen et al., 2024). BCAAs supply critical nitrogen sources for glutamate and γ-aminobutyric acid (GABA) production, both of which are strongly associated with depression. BCAA dysregulation may contribute to depression by influencing brain inflammation and oxidative stress (Tărlungeanu et al., 2016; Choi et al., 2024). This study observed reduced BCAA metabolic pathway activity in the depression group, indicating that this imbalance may interfere with neurotransmitter homeostasis and inflammatory regulation.

Pathways related to starch and sucrose metabolism, crucial for the energy supply of the gut microbiome and cellular growth were impaired in the depression group. These findings suggest weakened host-microbiome symbiosis, leading to energy insufficiency and reduced metabolic activity, potentially exacerbating depression.

## Conclusion and future direction

In conclusion, we identified four viral biomarkers, namely *s_Stenotrophomonas_virus_Pokken*, *g_Pokkenvirus*, *s_Dickeya_virus_AD1*, and *g_Alexandravirus*, that demonstrated robust diagnostic performance in depression, with AUCs exceeding 0.8 and strong intercorrelations. Functional annotation suggested that depression is associated with seven altered pathways, especially BCAAs biosynthesis and amino acid metabolism.

These results provide foundational evidence that the gut virome plays an important role in depression. However, the study’s limitations, including moderate sample size and metagenomic-based sequencing without viral particle enrichment, necessitate validation in larger, more diverse cohorts. This study did not integrate bacterial metagenomics. Future studies should integrate longitudinal virome dynamics, host transcriptomics, phage-host network analysis, and mechanistic studies to elucidate causality and advance the translation of viral biomarkers into clinical diagnostics.

## Data Availability

The data presented in the study are deposited in the China National Center for Bioinformation (CNCB) repository, under BioProject number PRJCA046198 and GSA accession number CRA030037.
