# Supplementary material for: Gut viral metagenomics identifies viral signatures and their role in depression
Source: Front Microbiol. 2025 Oct 3;16:1573851. doi: 10.3389/fmicb.2025.1573851 (PMC12531219; doi:10.3389/fmicb.2025.1573851)
Supplement: Supplementary file 1 [file Supplementary_file_1.docx]

**Gut viral metagenomics identifies viral signatures and their role in depression**

**Supplementary Information**

**Supplemental Fig. 1 Observed rarefaction at species (left), genus (middle), and family (right) levels**

**Supplemental Fig. 2** Stacked taxonomic classification of the virus catalog at the levels of phylum (A), class (B), and family (C).

**Supplemental Fig. 3** **Viral community composition analysis at** genus level (A) α-diversity: Shannon analysis (upper panel of A) and Simpson analysis (lower panel of A); (B) β diversity analysis: ANOSIM analysis (upper panel of B) and NMDS analysis (lower panel of B).

**Supplemental Fig. 4** Functional annotation of virome at KEGG level 2

Supplemental Table 1. Diagnostic potential of four viral signatures individually and in combination in testing models

| Model No. | viral signatures | AUC | cutoff | Sensitivity | Specificity |
| --- | --- | --- | --- | --- | --- |
| 1 | s_Stenotrophomonas_virus_Pokken | 0.767 | 0.275 | 0.5 | 1 |
| 2 | g_Pokkenvirus | 0.767 | 0.275 | 0.5 | 1 |
| 3 | s_Dickeya_virus_AD1 | 0.867 | 0.252 | 0.667 | 1 |
| 4 | g_Alexandravirus | 0.867 | 0.369 | 0.667 | 1 |
| 5 | s_Stenotrophomonas_virus_Pokken, g_Pokkenvirus | 0.767 | 0.275 | 0.5 | 1 |
| 6 | s_Stenotrophomonas_virus_Pokken, s_Dickeya_virus_AD1 | 0.8 | 0.252 | 0.667 | 1 |
| 7 | s_Stenotrophomonas_virus_Pokken, g_Alexandravirus | 0.867 | 0.367 | 0.667 | 1 |
| 8 | g_Pokkenvirus, s_Dickeya_virus_AD1 | 0.8 | 0.252 | 0.667 | 1 |
| 9 | g_Pokkenvirus, g_Alexandravirus | 0.867 | 0.367 | 0.667 | 1 |
| 10 | s_Dickeya_virus_AD1, g_Alexandravirus | 0.867 | 0.249 | 0.667 | 1 |
| 11 | s_Stenotrophomonas_virus_Pokken, g_Pokkenvirus, s_Dickeya_virus_AD1 | 0.8 | 0.252 | 0.667 | 1 |
| 12 | s_Stenotrophomonas_virus_Pokken,g_Pokkenvirus, g_Alexandravirus | 0.8 | 0.252 | 0.667 | 1 |
| 13 | s_Stenotrophomonas_virus_Pokken, s_Dickeya_virus_AD1, g_Alexandravirus | 0.867 | 0.367 | 0.667 | 1 |
| 14 | g_Pokkenvirus, s_Dickeya_virus_AD1, g_Alexandravirus | 0.8 | 0.249 | 0.667 | 1 |
| 15 | s_Stenotrophomonas_virus_Pokken, g_Pokkenvirus, s_Dickeya_virus_AD1, g_Alexandravirus | 0.8 | 0.249 | 0.667 | 1 |
